# Optoelectronic Evolution in Halogen-Doped Organic–Inorganic Halide Perovskites: A First-Principles Analysis

**DOI:** 10.3390/molecules28217341

**Published:** 2023-10-30

**Authors:** Cheng-Liang Xiao, Sicheng Liu, Xiao-Yan Liu, Yi-Ning Li, Peng Zhang

**Affiliations:** School of Space Science and Physics, Shandong University, Weihai 264209, China; chengliang_xiao@mail.sdu.edu.cn (C.-L.X.); liusicheng@mail.sdu.edu.cn (S.L.); liuxiaoyan@mail.sdu.edu.cn (X.-Y.L.); yiningli@mail.sdu.edu.cn (Y.-N.L.)

**Keywords:** solar cells, organic–inorganic hybrid perovskites, first-principles calculations, halide perovskites

## Abstract

Cl, Br, and I are elements in the halogen family, and are often used as dopants in semiconductors. When employed as dopants, these halogens can significantly modify the optoelectronic properties of materials. From the perspective of halogen doping, we have successfully achieved the stabilization of crystal structures in CH_3_NH_3_PbX_3_, CH_3_NH_3_PbI_3−x_Cl_x_, CH_3_NH_3_PbI_3−x_Br_x_, and CH_3_NH_3_PbBr_3−x_Cl_x_, which are organic–inorganic hybrid perovskites. Utilizing first-principles density functional theory calculations with the CASTEP module, we investigated the optoelectronic properties of these structures by simulations. According to the calculations, a smaller difference in electronegativity between different halogens in doped structures can result in smoother energy bands, especially in CH_3_NH_3_PbI_3−x_Br_x_ and CH_3_NH_3_PbBr_3−x_Cl_x_. The PDOS of the Cl-3p orbitals undergoes a shift along the energy axis as a result of variances in electronegativity levels. The optoelectronic performance, carrier mobility, and structural stability of the CH_3_NH_3_PbBr_3−x_Cl_x_ system are superior to other systems like CH_3_NH_3_PbX_3_. Among many materials considered, CH_3_NH_3_PbBr_2_Cl exhibits higher carrier mobility and a relatively narrower bandgap, making it a more suitable material for the absorption layer in solar cells. This study provides valuable insights into the methodology employed for the selection of specific types, quantities, and positions of halogens for further research on halogen doping.

## 1. Introduction

In 2009, Kojima et al. introduced a novel dye-sensitized material known as the organic–inorganic hybrid perovskite CH_3_NH_3_PbX_3_ (X = I, Br). This material is cost-effective and can be produced at temperatures that are comparatively lower. At that time, solar cells exhibited a maximum power conversion efficiency (PCE) of merely 3.81%, and exhibited high instability. This marked the emergence of organic–inorganic hybrid perovskite solar cells [1]. In the past decade, there has been a remarkable advancement in the power conversion efficiency (PCE) of perovskite solar cells [2,3,4,5,6,7,8,9,10]. In the field of computational research on organic–inorganic hybrid perovskite structures, in 2013, Mosconi et al. conducted first-principles calculations on CH_3_NH_3_PbX_3_ (X = I, Br, Cl) and found a blue shift in the absorption spectrum as the halogen X changes from I to Br to Cl. Furthermore, they observed that the band structure trends in line with the experimentally obtained perovskite bandgap [11]. Additionally, they found that within the CH_3_NH_3_PbI_2_X (X = Cl, Br) system, Cl atoms exhibit a preference for occupying the top position of the PbI_2_X_4_ octahedron, while Br atoms have the capability to occupy either the top or equatorial positions. In 2014, Giacomo et al. employed first-principles calculation methods to examine the influence of organic cations on the optoelectronic properties of synthesized organic–inorganic hybrid perovskites CH_3_NH_3_PbX_3_ (X = I, Br, Cl). They found that in two-dimensional situations, organic molecules have a minimal impact on the band structure of perovskites, but they can alter the shape and orbital positions of the band edges [12]. In 2016, Pan et al. also used first-principles calculations to investigate the electronic structure and orbital compositions of the conduction and valence bands of CH_3_NH_3_PbX_3_ (X = I, Br, Cl). It was discovered that CH_3_NH_3_PbI_3_ can generate and transport more electrons compared to the other two structures [13]. As research has progressed, doping halogen elements has been verified as an effective strategy for modifying the physical properties of materials [14,15,16,17,18,19,20,21,22,23,24,25]. In 2019, Pramchu et al. applied density functional theory to investigate the impact of Cl and Br doping on the surface of CH_3_NH_3_PbI_3_ perovskites and their influence on structural stability. They found that Cl and Br dopants with higher binding energy to the surrounding atoms can prevent the deterioration of the perovskite. It was claimed that adding Cl/Br to MAPbI_3_ could overcome instability issues and enhance the efficiency of perovskite solar cells based on MAPbI_3_ [26]. While there have been numerous studies of halogens, the impact of the doping levels of I, Br, and Cl, and their impacts on the optoelectronic properties of organic–inorganic hybrid perovskite materials remain unknown. This includes adjusting the material’s bandgap through halogen doping, enhancing the material’s thermal stability and chemical stability, improving charge migration performance within the material, influencing crystal lattice parameters and structure, altering the material’s absorption characteristics at specific wavelengths, or addressing inherent defects within the material. Based on this, we conducted calculations on the established hybrid perovskite crystal structures, utilizing first-principles calculation methods to derive the electronic structure and optoelectronic properties of perovskite crystals in different systems. We evaluated the computed findings and investigated the regularities of the influence of halogen atom doping at different positions and in different quantities on the structure and properties of hybrid organic–inorganic perovskite materials. The observed results show that doping halogen elements with closer electronegativity and atomic radii can yield a more stable structure, resulting in a larger calculated curvature radius of the energy band. Due to the variation in doping elements, the PDOS of the Cl 3p orbitals experiences a shift along the energy axis, leading to a blue shift in the absorption spectrum and a consequent narrowing of the absorption range when doped with more electronegative elements.

## 2. Results and Discussion

### 2.1. Geometric Properties

**Figure 1:**
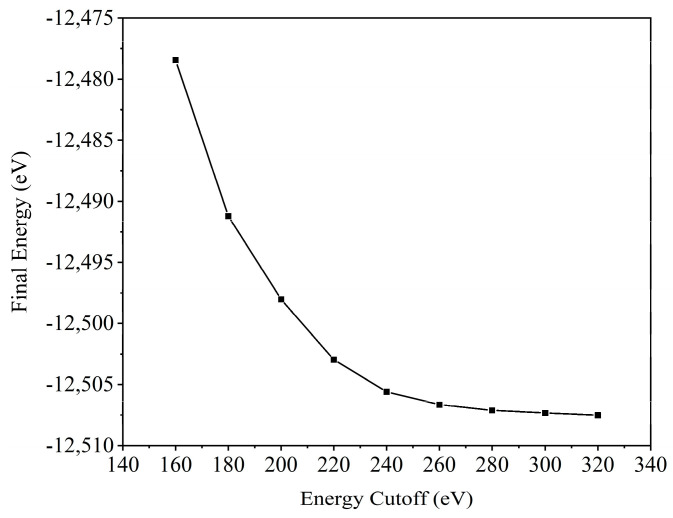
Test results for convergence of structural models.

In comparison with the original CH_3_NH_3_PbX_3_ structure, the introduction of halogen atoms as dopants in the organic–inorganic hybrid perovskite structures CH_3_NH_3_PbI_3−x_Cl_x_, CH_3_NH_3_PbI_3−x_Br_x_, and CH_3_NH_3_PbBr_3−x_Cl_x_ causes distortions and a reduction in symmetry. This has a major impact on the bonding and properties of atoms within the crystal. Simulation calculations of the crystal structure play a crucial role in the study of such perovskites. The choice of an appropriate crystal structure model has a significant impact on the accuracy and efficiency of the calculations. Before calculating the original structure using first-principles methods, we tested the structural model’s convergence. The convergence test results are displayed in Figure 1. Considering both accuracy and efficiency, we selected a cutoff energy of 280 eV for the computations.

**Figure 2:**
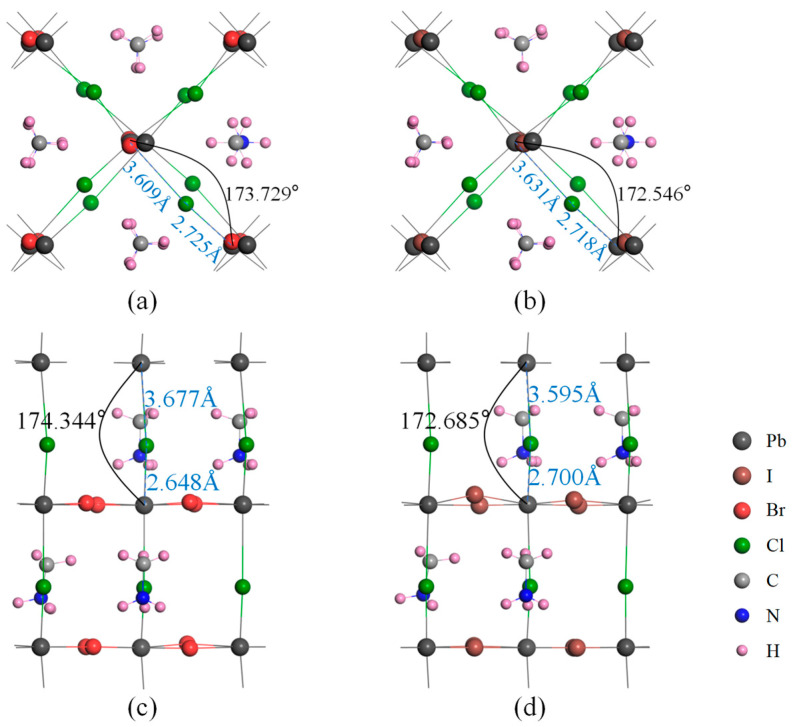
Optimized geometric structures of CH_3_NH_3_PbI_3−x_Cl_x_ and CH_3_NH_3_PbBr_3−x_Cl_x_ perovskites. (**a**,**b**) are the top views of CH_3_NH_3_PbBrCl_2_ and CH_3_NH_3_PbICl_2_, respectively. (**c**,**d**) are the side views of CH_3_NH_3_PbBr_2_Cl and CH_3_NH_3_PbI_2_Cl, respectively. Numbers in all figures represent the bond lengths and bond angles, respectively.

Using first-principles calculations, we optimized the crystal structures of all materials. For the optimization of CH_3_NH_3_PbI_3_, the largest change observed in bond length was 0.070 Å, while the largest change in bond angle was 2.307° compared with the characteristics of the same structures in reality. In addition, we examined the bond angles and lengths of the Pb-Cl-Pb bonds in the optimized structures of CH_3_NH_3_PbI_3−x_Cl_x_ and CH_3_NH_3_PbBr_3−x_Cl_x_, as shown in Figure 2. It was found that the different doping elements had little effect on the bond length of Pb-Cl, but the Pb-Cl-Pb bond angle in the I-doped structure was smaller than that in the Br-doped structure at both octahedral vertices and equatorial positions. To analyze this phenomenon, we need to consider the differences between I and Br, including atomic size and steric repulsion, electronegativity, and electric field effects. When the I atom approaches the Pb-Cl-Pb structure, its large volume will cause steric repulsion with the nearby Pb-Cl-Pb, which will cause the Pb-Cl-Pb structure to deform to avoid contact with the I atom, resulting in the deviation of its bond angle from 180 degrees. In contrast, the steric repulsion caused by the smaller Br atom is relatively weak, so the Pb-Cl-Pb bond angle in the presence of Br is closer to 180 degrees. In addition, the electronegativity of Br is closer to that of Cl. The interaction between Br and the electron cloud of the Pb-Cl-Pb bond will cause the electrons in the bond to redistribute, resulting in a bond angle closer to 180 degrees. The lower electronegativity of I will not cause a significant electric field effect on the Pb-Cl-Pb bond angle, resulting in a deviation from 180 degrees. The closer the bond angle is to 180 degrees, the more balanced the repulsive force between the electrons on the central atom, and the more symmetrical the crystal structure becomes. This symmetry contributes to the excellent stability of the CH_3_NH_3_PbBr_3−x_Cl_x_ structures.

### 2.2. Electronic Properties

The condition for a material to generate electron-hole pairs is that the incident solar light ħω on the material surface is greater than the bandgap energy. Each pair can release the maximum energy that can be converted into short-circuit current, which is closely related to the bandgap width of the material. When the bandgap is greater, each electron-hole pair can release more energy. However, only a portion of ħω greater than the bandgap can be absorbed and excited into electron-hole pairs. In addition, although narrow bandgap materials are highly capable of generating electron-hole pairs, most of the energy is wasted due to the conversion of carriers and lattice collisions into heat energy, resulting in the very little energy that can be released by electron-hole pairs. Therefore, the value of the bandgap is an important parameter that affects the performance of solar cells. When the bandgap is roughly 2.5–2.6 eV, which is slightly less than the peak of the solar energy distribution, an optimal state is reached [27,28]. We calculated the band structures and bandgap widths of the optimized geometries of various systems using first-principles calculations, as presented in Table 1.

**Figure 3:**
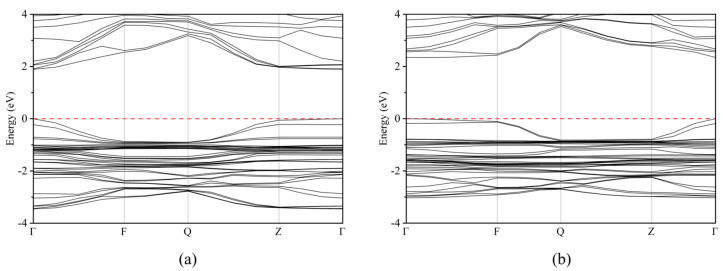
Band structure of: (**a**) CH_3_NH_3_PbI_2_Cl, (**b**) CH_3_NH_3_PbICl_2_ perovskites.

According to the calculation results mentioned above, doping with I, Br, and Cl will cause particular modifications to the structure’s lattice characteristics. Additionally, the bandgap of different structures will exhibit significant variations. The bandgap values of CH_3_NH_3_PbI_3_, CH_3_NH_3_PbI_3−x_Cl_x_, and CH_3_NH_3_PbCl_3_ structures were found to be 1.730 eV, 1.895 eV, 2.343 eV, and 3.183 eV, respectively. Compared with the reported values of 1.57 eV for CH_3_NH_3_PbI_3_ and 3.02 eV for CH_3_NH_3_PbCl_3_, the calculated values are slightly higher, with an error less than 10%, but they are in good agreement with the actual values, proving that our simulation calculations are instructive [29]. Based on the calculated bandgap size, CH_3_NH_3_PbBrCl_2_ and CH_3_NH_3_PbCl_3_ have a bandgap greater than 3 eV, making it difficult to excite, and are not suitable as light-absorbing materials for solar cells. The other systems are suitable as light-absorbing layers for solar cells. From the band structure diagram below, it can be seen that the Fermi level of all nine systems is located near the valence band, and the maximum value of the valence band top and the minimum value of the conduction band bottom appear at the same k point, indicating the characteristics of a direct bandgap p-type semiconductor, as shown in Figure 3 (with CH_3_NH_3_PbI_3−x_Cl_x_ as an example). Compared with indirect bandgap semiconductors, direct bandgap semiconductors have higher photoelectric conversion efficiency because electrons can directly transition to the conduction band upon energy absorption.

**Figure 4:**
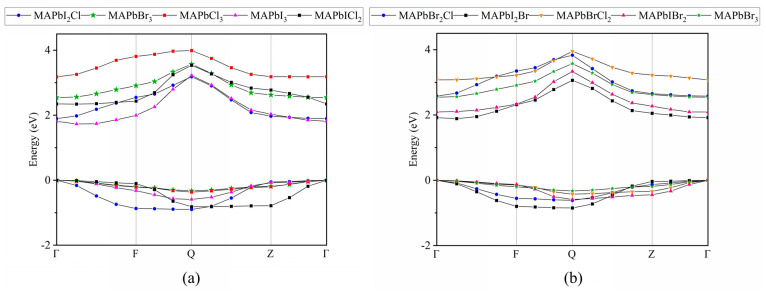
The valence band maximum and conduction band minimum of: (**a**) CH_3_NH_3_PbX_3_ (X = I, Br, Cl) and CH_3_NH_3_PbI_3−x_Cl_x_, (**b**) CH_3_NH_3_PbBr_3_, CH_3_NH_3_PbI_3−x_Br_x_ and CH_3_NH_3_PbBr_3−x_Cl_x_ perovskites.

In addition, the structures of the valence band maximum (VBM) and the conduction band minimum (CBM) of the nine systems were analyzed, as shown in Figure 4. From Figure 4a, it can be seen that the conduction band minimum and valence band maximum of CH_3_NH_3_PbCl_3_ and CH_3_NH_3_PbBr_3_ structures are flatter than those of other systems, making it easier for the conduction band minimum to accept electrons transitioning from the valence band, forming carriers. Therefore, this flat structure makes electron transition faster and more effective, making it more advantageous as a photoelectric conversion material. However, the energy band curve of the conduction band minimum in the CH_3_NH_3_PbI_3−x_Cl_x_ system exhibits a significant curvature, which results in a higher degree of curvature; As a result, it is less likely to accept electrons transitioning from the valence band. In the Cl-doped system, the bandgap lies between CH_3_NH_3_PbCl_3_ and CH_3_NH_3_PbI_3_. The bandgap of CH_3_NH_3_PbI_2_Cl exhibits a high degree of similarity to that of CH_3_NH_3_PbI_3_. However, the valence band maximum and the conduction band minimum of CH_3_NH_3_PbI_2_Cl and CH_3_NH_3_PbICl_2_ structures are the sharpest, which makes them unsuitable for facilitating rapid electron transition.

From Figure 4b, we used the flattest CH_3_NH_3_PbBr_3_ as a reference point to assess the flatness of the CH_3_NH_3_PbI_3−x_Br_x_ and CH_3_NH_3_PbBr_3−x_Cl_x_ systems. We discovered that the flatness of these two systems is comparable to that of CH_3_NH_3_PbBr_3_. 

In the periodic table, the atomic radii of Cl, Br, and I gradually increase. Because the atomic radius of Br is between Cl and I, the spatial changes it causes in the lattice are moderate. This means that the lattice distortion of CH_3_NH_3_PbI_3−x_Br_x_ and CH_3_NH_3_PbBr_3−x_Cl_x_ is smaller than that of CH_3_NH_3_PbI_3−x_Cl_x_. The reduced lattice distortion helps to obtain a more stable and smooth energy band. Different electronegativities will also affect the bond strength between halogen atoms and Pb atoms. The electronegativity difference between I and Br and between Br and Cl is smaller than that between I and Cl, making the chemical bonds formed by CH_3_NH_3_PbI_3−x_Br_x_ and CH_3_NH_3_PbBr_3−x_Cl_x_ closer to CH_3_NH_3_PbBr_3_, which helps to obtain a smoother energy band structure. In addition, the energy levels of different halogen p-orbitals vary, which will impact their overlap with the Pb 6s orbital. When the Pb 6s orbital hybridizes with the p orbitals of different halogens, the resulting hybrid orbitals will also be different, which will further affect the band structure. This will be further discussed in the analysis of PDOS later on.

When the electronegativity and atomic radius differences of halogen elements are smaller, the distribution of the electric field in the crystal structure is more uniform, and the arrangement of atoms is more closely packed and orderly. This reduces the scattering effects (including lattice scattering and impurity scattering) experienced by electrons during their motion. As a result, carriers experience less scattering, leading to a faster migration rate and, consequently, an increase in the material’s electrical conductivity. This is particularly crucial in semiconductors and photovoltaic materials. In solar cells, rapid carrier migration enhances the efficiency of the cells by reducing the probability of carrier recombination before reaching the electrodes, thereby minimizing energy loss.

**Figure 5:**
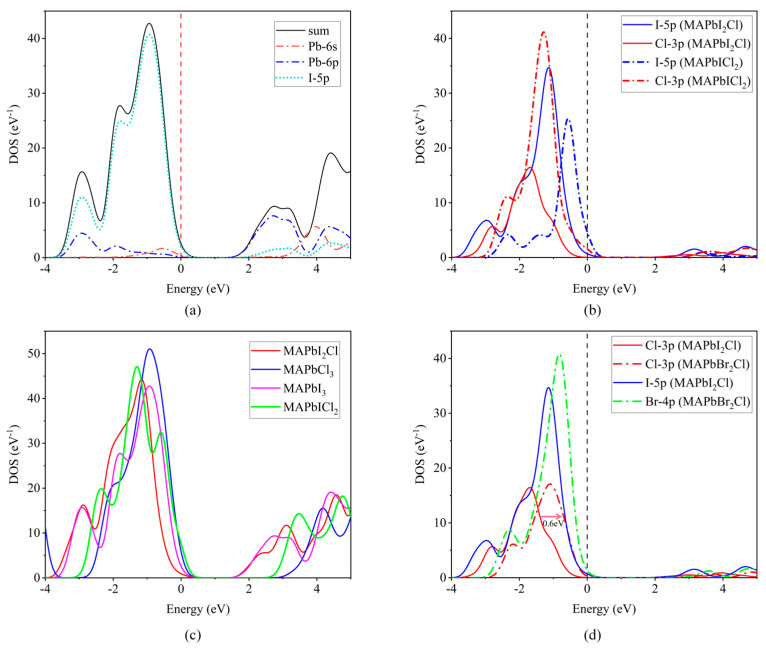
(**a**) DOS and PDOS of CH_3_NH_3_PbI_3_ perovskites. (**b**) PDOS of the Cl 3p orbitals and the I 5p orbitals of CH_3_NH_3_PbI_3−x_Cl_x_ perovskites. (**c**) DOS of CH_3_NH_3_PbI_3_, CH_3_NH_3_PbCl_3_ and CH_3_NH_3_PbI_3−x_Cl_x_ perovskites. (**d**) PDOS of CH_3_NH_3_PbI_2_Cl and CH_3_NH_3_PbBr_2_Cl perovskites.

By conducting first-principles calculations, it is feasible to attain the density of states (DOS) for each system, as well as the partial density of states (PDOS) of each atom within those systems. The valence band maximum is mainly contributed by the presence of halogens, while the contribution of Pb is predominantly responsible for the conduction band minimum. The valence band maximum is obtained from the halogen 5p state, while the conduction band minimum is obtained from the Pb 6s and 6p states. There is a relatively weak covalent interaction between Pb and halogens. The organic cation MA+ does not contribute to the valence band maximum or conduction band minimum, as shown in Figure 5a. In the systems CH_3_NH_3_PbI_2_Cl and CH_3_NH_3_PbICl_2_, the valence band maximum is jointly obtained from the I 5p state and the Cl 3p state, but the peak intensities of the two atoms are different. In CH_3_NH_3_PbI_2_Cl, the number of electrons on I is greater than that of Cl, indicating a stronger interaction between I and Pb compared to Cl. The opposite is true for CH_3_NH_3_PbICl_2_. This indicates that as the number of Cl substitutions increases, the contribution of Cl gradually strengthens, as shown in Figure 5b. We compare the total density of states diagrams of different systems, taking CH_3_NH_3_PbI_3_, CH_3_NH_3_PbCl_3_, and CH_3_NH_3_PbI_3−x_Cl_x_ as examples. The density of states of the four systems exhibit similarities, and their molecular orbital bonding types are basically the same, with only minor variations in position. This indicates that the change of halogens has little effect on the overall orbital bonding, as shown in Figure 5c.

Now, let us focus on the issue of band smoothness that we discovered earlier. We take CH_3_NH_3_PbI_2_Cl and CH_3_NH_3_PbBr_2_Cl as examples and calculate the PDOS of their respective halogens, as shown in Figure 5d. Under the same doping amount, we found that the PDOS of the Cl 3p orbitals (two red lines) of CH_3_NH_3_PbI_2_Cl and CH_3_NH_3_PbBr_2_Cl shifted toward the Fermi level along the energy axis by 0.6 eV, due to the electronegativities of I, Br, and Cl being 2.66, 2.96, and 3.16, respectively. This difference in electronegativity implies that when adjacent to I, Cl undergoes a relatively small electric field effect. However, when adjacent to Br, it experiences an increased electric field effect, which results in different degrees of hybridization between the p orbitals of I and Br with the Cl 3p orbital, leading to a change in the energy level of the Cl 3p orbital. The difference in electronegativity also causes different extents of charge transfer from I or Br to Cl, which changes the charge state of the Cl atom, thereby affecting the energy level of its 3p orbital. In addition, the atomic radius of I is larger than that of Br, and the spatial relationship and interatomic distance in the two lattices are different. This difference also leads to a change in the strength of interaction between atoms.

In conclusion, due to the combined effects of electronegativity, atomic size, orbital overlap, hybridization, and charge transfer, the PDOS of the Cl 3p orbitals undergoes a shift along the energy axis.

### 2.3. Effective Masses

Carrier mobility, which is typically closely linked to the effective mass of electrons, is a crucial parameter for evaluating the potential for utilization in optoelectronic devices. In general, semiconductors with a small effective mass of carriers exhibit high carrier mobility. To analyze the mobility and transport properties of organic–inorganic halide perovskites, we focused on their effective masses. We observed that the effective mass of electrons in CH_3_NH_3_PbX_3_ (X = I, Br, Cl) decreases sequentially from X = I to Br to Cl, as presented in Table 2. However, no clear pattern was found in other doped structures, which is attributed to the chosen path and the distribution of doped elements. On our calculated G-F path, the effective mass of electrons and holes is influenced by factors such as the optimized lattice structure, atomic species, chemical bond energies, impurities and defects, and electron–electron interactions. However, by observing the table data, we can still see that the electron effective mass of CH_3_NH_3_PbBr_2_Cl is the smallest along the G-F path. This unique structure can effectively enhance carrier mobility, allowing electrons or holes to move faster and reduce scattering effects. The overall conductivity of the material will also increase. Meanwhile, it can increase the response speed of the carrier. When applied to optoelectronic devices, the carrier can respond faster to external stimuli.

### 2.4. Absorption Spectrum

**Figure 6:**
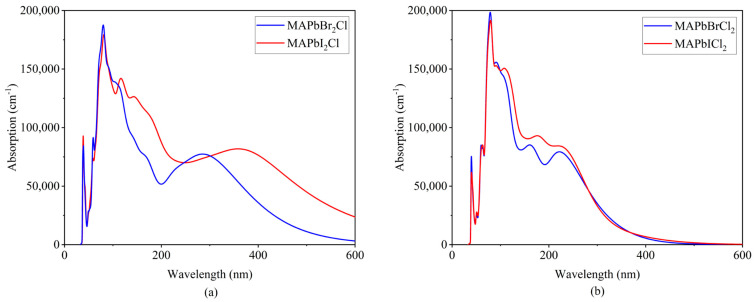
Absorption coefficient graphs of: (**a**) CH_3_NH_3_PbBr_2_Cl and CH_3_NH_3_PbI_2_Cl structures, (**b**) CH_3_NH_3_PbBrCl_2_ and CH_3_NH_3_PbICl_2_ structures.

The absorption spectrum reflects the material’s ability to absorb light in different wavelength bands. Therefore, analyzing the absorption spectrum can provide insights into the appropriate systems to be used as absorbing layers. By calculating the optical properties using first principles, we can obtain maps of the optical absorption coefficient for different systems. The optical absorption coefficient is directly proportional to the absorption spectrum. Therefore, the absorption spectrum can also be analyzed based on the absorption coefficient map. We analyzed the absorption spectra of CH_3_NH_3_PbI_3−x_Cl_x_ and CH_3_NH_3_PbBr_3−x_Cl_x_ and found that the absorption range of CH_3_NH_3_PbBr_3−x_Cl_x_ is narrower and shifted toward shorter wavelengths, as shown in Figure 6. This is similar to the findings of Mosconi et al. in 2013, where the organic–inorganic hybrid perovskites CH_3_NH_3_PbX_3_ (X = I, Br, Cl) showed a blue shift in the absorption spectrum from X = I to Br to Cl [11]. This indicates that doping with more electronegative elements can also achieve a blue shift in the absorption spectrum. Br has higher electronegativity compared to I, leading to an increased bandgap width. A wider bandgap means that photons need higher energy, i.e., shorter wavelengths, to excite electrons from the valence band to the conduction band. Irregularities and defects in the crystal structure can broaden the absorption spectrum. CH_3_NH_3_PbBr_3−x_Cl_x_ reduces such irregularities compared to CH_3_NH_3_PbI_3−x_Cl_x_, resulting in a narrower absorption spectrum. Although a narrower spectrum allows for higher resolution and sensitivity in the same spectral range, it can limit the efficiency of applications that require broad-spectrum absorption. In conclusion, it is important to determine its advantages and disadvantages based on specific applications and requirements. Further research can continue to modify the doping ratio of Br to achieve ideal performance.

## 3. Simulation Strategy

**Figure 7:**
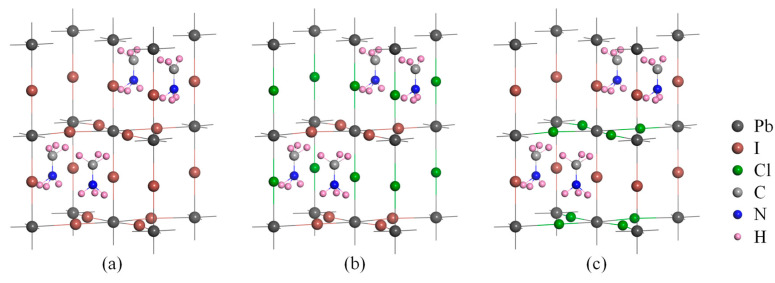
The geometric structures of: (**a**) CH_3_NH_3_PbI_3_, (**b**) CH_3_NH_3_PbI_2_Cl doped at the octahedral top, (**c**) CH_3_NH_3_PbICl_2_ doped at the equatorial position.

The CASTEP module is a first-principles calculation module based on density functional theory. Based on previous study findings and experimental comparisons, the GGA-PW91 exchange–correlation energy approximation method was selected, and the ultrasoft pseudopotential method was used for geometric optimization and calculation of relevant photoelectric properties [30,31,32,33,34,35,36,37]. Since the initial structure reported in the literature contains 48 atoms [38,39,40,41], as shown in Figure 7a, after convergence testing (detailed in the above text), considering the efficiency and accuracy of the calculations, a plane cutoff energy of 280 eV and a 3 × 3 × 2 k-point mesh were selected for the calculations. After obtaining the stable crystal structure of CH_3_NH_3_PbX_3_ (X = I, Br, Cl), different elements other than X were doped at the octahedral top and equatorial positions of the original perovskite CH_3_NH_3_PbX_3_ crystal structure, resulting in two different CH_3_NH_3_PbI_3−x_Cl_x_, CH_3_NH_3_PbI_3−x_Br_x_, and CH_3_NH_3_PbBr_3−x_Cl_x_ structures, totaling six, as shown in Figure 7b,c (with CH_3_NH_3_PbI_3−x_Cl_x_ as an example). The same method was used to calculate the geometric and electronic structures of these nine structures.

By utilizing the CASTEP program, geometric optimization and electronic structure calculations on the organic–inorganic hybrid perovskite can be conducted. This enables the acquisition of several parameters, such as band structure, density of states, charge distribution, and absorption spectra of the material’s crystal structure. Through the process of calculation, it is feasible to assess the impact of halogen atoms on the structure and properties of hybrid perovskites.

## 4. Conclusions

Based on our first-principles calculations, the crystal structure of CH_3_NH_3_PbBr_3−x_Cl_x_ is more stable compared to CH_3_NH_3_PbI_3−x_Cl_x_. Additionally, the bandgap of CH_3_NH_3_PbBr_2_Cl makes it suitable as a light-absorbing material for solar cells. The Cl-3p orbital PDOS in CH_3_NH_3_PbI_2_Cl shifts toward the Fermi level along the energy axis compared to CH_3_NH_3_PbBr_2_Cl, due to doping with halogen elements that have similar electronegativity and atomic radii. The band structures of CH_3_NH_3_PbI_3−x_Br_x_ and CH_3_NH_3_PbBr_3−x_Cl_x_ are smoother, making them more receptive to transition electrons compared to CH_3_NH_3_PbI_3−x_Cl_x_. CH_3_NH_3_PbBr_2_Cl has the smallest effective mass of electrons along the G-F path, resulting in high carrier mobility, strong conductivity, fast response, and beneficial properties for optoelectronic devices. Doping with elements of higher electronegativity can also achieve a blue shift in the absorption spectrum.

The optoelectronic performance of the CH_3_NH_3_PbBr_2_Cl system is superior to other systems, with a more stable structure. Electrons in this system can undergo transitions more rapidly and efficiently, and the bandgap is close to the optimal value, making it suitable as a light-absorbing material for solar cell layers. Overall, we provide a thorough analysis of the band structure, density of states, partial density of states, and effective mass of different systems. We emphasize the importance of considering the electronegativity of elements in predicting and explaining doping effects. Our investigations also reveal the consistent changes in optoelectronic performance that result from doping different elements. These provide important insights for further refinement of doping research and serve as a valuable guide for solar cell design.

## Figures and Tables

**Table 1:** Geometrically optimized bandgap for different structure types. (MA = CH_3_NH_3_).

Structure	Bandgap (eV)	Structure	Bandgap (eV)	Structure	Bandgap (eV)
MAPbI_3_	1.730	MAPbBr_3_	2.544	MAPbCl_3_	3.183
MAPbI_2_Cl	1.895	MAPbBr_2_Cl	2.578	MAPbI_2_Br	1.888
MAPbICl_2_	2.343	MAPbBrCl_2_	3.076	MAPbIBr_2_	2.093

**Table 2:** Effective carrier masses in different structure types of organic–inorganic halide perovskites (MA = CH_3_NH_3_).

Structure Type	m_e_	m_h_	Structure Type	m_e_	m_h_
MAPbI_3_	0.643 m_0_	−0.514 m_0_	MAPbI_2_Cl	0.265 m_0_	−0.207 m_0_
MAPbBr_3_	0.445 m_0_	−0.866 m_0_	MAPbICl_2_	1.692 m_0_	−1.642 m_0_
MAPbCl_3_	0.269 m_0_	−0.816 m_0_	MAPbBr_2_Cl	0.223 m_0_	−0.316 m_0_
MAPbI_2_Br	0.372 m_0_	−0.216 m_0_	MAPbBrCl_2_	1.125 m_0_	−1.181 m_0_
MAPbIBr_2_	0.688 m_0_	−1.308 m_0_	Path: G-F; m_0_ is the mass of a free electron		

## Data Availability

Data are available from the authors.
